# On-Board Real-Time Trajectory Planning for Fixed Wing Unmanned Aerial Vehicles in Extreme Environments

**DOI:** 10.3390/s19194085

**Published:** 2019-09-21

**Authors:** Ben Schellenberg, Tom Richardson, Arthur Richards, Robert Clarke, Matt Watson

**Affiliations:** 1 Department of Aerospace Engineering, University of Bristol, Bristol BS8 1TR, UK; Thomas.Richardson@bristol.ac.uk (T.R.); Arthur.Richards@bristol.ac.uk (A.R.); Robert.Clarke@bristol.ac.uk (R.C.); 2 Bristol Robotics Laboratory, University of Bristol, Bristol BS16 1QY, UK; 3 School of Earth Sciences, University of Bristol, Bristol BS8 1RJ, UK; Matt.Watson@bristol.ac.uk

**Keywords:** UAV path planning, UAV navigation, environmental monitoring

## Abstract

A team from the University of Bristol have developed a method of operating fixed wing Unmanned Aerial Vehicles (UAVs) at long-range and high-altitude over Volcán de Fuego in Guatemala for the purposes of volcanic monitoring and ash-sampling. Conventionally, the mission plans must be carefully designed prior to flight, to cope with altitude gains in excess of 3000 m, reaching 9 km from the ground control station and 4500 m above mean sea level. This means the climb route cannot be modified mid-flight. At these scales, atmospheric conditions change over the course of a flight and so a real-time trajectory planner (RTTP) is desirable, calculating a route on-board the aircraft. This paper presents an RTTP based around a genetic algorithm optimisation running on a Raspberry Pi 3 B+, the first of its kind to be flown on-board a UAV. Four flights are presented, each having calculated a new and valid trajectory on-board, from the ground control station to the summit region of Volcań de Fuego. The RTTP flights are shown to have approximately equivalent efficiency characteristics to conventionally planned missions. This technology is promising for the future of long-range UAV operations and further development is likely to see significant energy and efficiency savings.

## 1. Introduction

**Figure 1:**
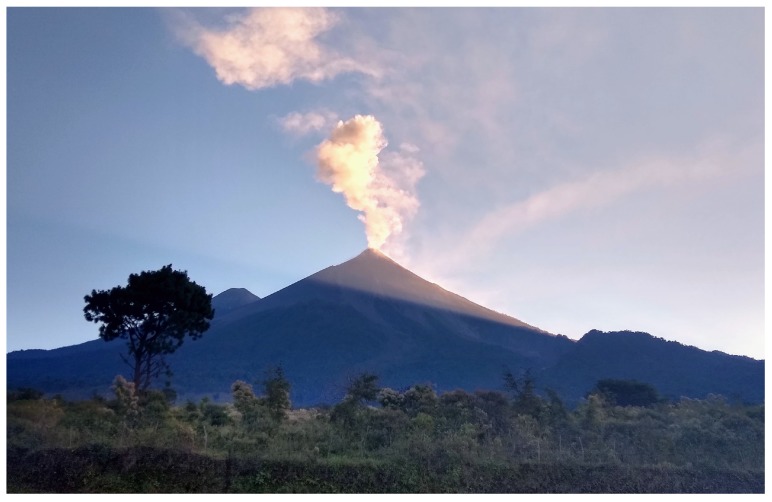
Sunrise over Volcań de Fuego, from the Ground Control Station.

A fixed wing Unmanned Aerial Vehicle (UAV) platform has been developed by a team from the University of Bristol for flight over Volcán de Fuego, Guatemala, in order to collect ash samples and atmospheric measurements from within the volcanic plume. Volcán de Fuego (Figure 1, hereafter Fuego) is an active volcano with small explosions up to four times per hour and larger events every 6–8 weeks. The operational environment around Fuego is extreme; it is common for the aircraft to encounter strong winds at altitude, with high levels of atmospheric turbulence, during missions to the summit region. In addition to highly variable wind, aircraft also experience large temperature variations and unpredictable, rapidly developing meteorological clouds. Although full permissions exist for UAV flights in an extended area around Fuego, other air traffic occasionally operates in the area around the Ground Control Station (GCS), where take-off occurs, at times requiring intervention to maintain separation.

Missions have typically been flown according to a predetermined waypoint plan, which often results in wasted energy, reducing the available time at the target location. Unexpected conditions are typically handled by aborting the flight and refining the mission for the next flight. A reliable method of reaching the target location, while minimising energy use and taking into account the current atmospheric conditions, would increase the time spent collecting useful scientific data. A Real-Time Trajectory Planner (RTTP) implemented on-board the UAV would allow mission plans to be updated on the fly, with future applications incorporating sense-and-avoid technology and aircraft performance optimisation. A full solution would sense these changes in the aircraft performance, environment, or air traffic, and generate a new mission plan which minimises unnecessary energy wastage from the current location to the mission target point. Upon arriving at a new operating site, a significant amount of time is often spent developing a suitable and efficient conventional flight plan. With a full RTTP in place, the time taken from arrival at a new site to flying an efficient and successful mission would be reduced significantly.

While there are numerous motion and path planning algorithms and optimisations in existence, few have been considered with three-dimensional UAV implementations in mind. There are no published cases of real-time trajectory optimisation taking place on-board a UAV mid-flight. Optimisation methods typically consist of an algorithm to minimise some ‘cost function’. Cost functions can have single or multiple objectives and in the context of UAV motion planning it may, for example, include a function of path length and proximity to terrain. Certain limits can also be imposed which form ‘hard’ constraints on the problem, for example separation from an obstacle being positive (i.e., never impacting it), or the continuity of the path being maintained (to ensure it is flyable). The cost function implemented for the work in this paper will be discussed in Section 3.3.

Autonomy in UAVs follows many definitions, usually involving an autopilot unit controlling the actuators and throttle such that controlled flight is achieved. NATO define four levels to classify the autonomy of a UAV [1]:

- **Level 1, Remotely Controlled System:** System reactions and behaviour depend on operator input,
- **Level 2, Automated System:** Reactions and behaviour depend on fixed built-in functionality,
- **Level 3, Autonomous Non-Learning System:** Behaviour depends on fixed built-in functionality or upon a fixed set of rules that dictate system behaviour (goal-directed reaction and behaviour),
- **Level 4, Autonomous learning system with the ability to modify rules defining behaviours:** Behaviour depends upon a set of rules that can be modified for continuously improving goal directed reactions and behaviours within an overarching set of inviolate rules/behaviours.

For example, a UAV following a set mission plan can be classified as Level 2 or Level 3. However, generating a mission plan on-board and in real-time could be classified as Level 3 or Level 4. The benefits of real-time flight path generation include dynamic obstacle avoidance and aircraft performance optimisation. In cases where tracking or searching flight is required, the aircraft could adjust its flight pattern in real-time according to the results of the search. Letheren et al. have flown multi-rotor UAVs with a route generated in real-time by a bio-inspired algorithm intended to find the source of a forest fire. However, this was limited to 2D within Visual Line of Sight (VLOS), with the computation of the path carried out on the GCS [2]. Where UAVs are required to operate over longer ranges, extending into Beyond Visual Line of Sight (BVLOS) [3] operations, the telemetry link to the aircraft typically has lower bandwidth than VLOS operations. This has implications for the feasibility of performing the necessary computation on the GCS, providing motivation to move the planning computation on-board the vehicle.

In this paper, we present a functioning real-time trajectory planner, situated on-board the aircraft, which has been flown in the real world. While the vehicle’s progress was carefully monitored, it was acting entirely under the control of the RTTP. The remote pilot only intervened to ensure that the operations remained safe. The system presented in this paper effectively increased the level of automation of the UAV in question at the time of testing.

## 2. Background

At the core of the work presented here is the trajectory planning algorithm and its real-world implementation. A number of teams have investigated fixed-wing UAV path planning; however, none of this previous work involved real world testing or use. Some related real world testing has taken place with multi-rotor UAVs [2]; however, these aircraft are innately different to fixed-wing UAVs as they have the ability to stop moving to buy computational time. The application presented in this paper requires route solutions that span a large space, involving BVLOS operation.

The practical use of successful 3D trajectory optimisation is a natural area of development in the UAV community, increasing UAV flight performance, developing UAV automation, and increasing UAV operators’ efficiency. The application of reliable real-time path planning methods would allow UAV teams to save the time spent developing efficient and effective flight plans in order to complete specific tasks. It is also of interest to the air traffic conflict detection and resolution (CDR) community [4], both for integration of UAVs into existing airspace, and development of UAV-specific air traffic management.

Trajectory optimisation has been an area of interest for a long time, with Betts’ survey of numerical methods for trajectory optimisation dating back to 1998 [5]. Betts formulates the problem generically, including various methods of problem set-up, before discussing a variety of solution methods. Various shooting and collocation methods are discussed, and compared briefly to genetic algorithms and simulated annealing; randomness in these methods makes them attractive to certain practical cases. While Betts considers trajectory optimisation in a more general sense, Hoy et al. consider algorithms for robotic navigation through cluttered environments; a survey that is more relevant to the UAV case, despite just considering 2D cases [6]. Path planning algorithms such as rapidly exploring random trees [7], graph search algorithms [8,9], and artificial potential field methods [10] are mentioned, as well as evolutionary algorithms, simulated annealing, and particle swarm optimisation. Sensor based techniques, such as boundary following, are discussed; however, they are not suitable to the UAV problem presented in this paper. The world-space in the UAV problem is large and the aim is not to specifically avoid obstacles but rather to achieve an efficient flight to the target location, avoiding any obstacles that may be present.

Patle et al. review path planning strategies for mobile robots in their 2019 paper; however, again this is restricted to 2D [11]. A significant number of recent, biologically inspired, methods are discussed; for example, the ant colony optimisation [12] and the artificial bee colony algorithm [13]. Despite reviewing papers for a 2D application, suitability to 3D workspaces is considered. Methods that are listed as suitable for aerial navigation include genetic algorithms and particle swarm optimisation. A number of established optimisation methods are discussed below, including pertinent applications to path planning and UAVs.

### 2.1. Simulated Annealing

Simulated Annealing (SA) is a stochastic optimisation method that was originally presented by Davis [14], but developed and implemented for the path planning of robots by Martínez-Alfaro and Gómez-García [15], with a fuzzy logic control applied to keep the robots tracking the path. SA takes its inspiration from the process of annealing used to tailor crystal growth in metals by controlling their temperature. The principle of SA is a series of iterative searches, changing the configuration of the solution each time by (usually small) variable amounts. The magnitude of these changes can be controlled by a decaying ‘temperature’. The resulting configuration is evaluated for cost. The change in the cost function relative to the prior configuration is tested against some acceptance function and, should it pass this test, the changes are accepted. One of the key characteristics of SA is the existence of a decaying temperature in the acceptance function, so exploitation is more preferable to exploration as the number of iterations increases. SA was selected for initial testing but not used in the final implementation due to comparatively poor performance in 3D applications.

### 2.2. Evolutionary and Genetic Algorithms

Another form of stochastic optimisation technique is the Genetic Algorithm (GA) [14], widely used for applications such as the design of minimal phase digital filters and surface grinding processes [16]. GAs simulate the process of genetic evolution found in nature, and so the terminology associated with the process is therefore bio-inspired; the external characteristics (phenotype) are determined by the configuration of genes (genotype) [17]. Individuals who adapt poorly to the environment tend to be eliminated from the population while fitter individuals survive, and, as generations pass, processes such as mutations and recombinations ensure a positive evolution occurs. Within the optimisation method, each individual is represented through a series of bits, integers or numbers (‘chromosomes’) thereby making up the information within a genotype. Classical GAs use binary coding for the representation of the genotype.

Nikolos et al. present a form of GA called the Evolutionary Algorithm (EA), in the context of UAV path planning [17]. They describe the EA method in detail, and use B-splines to model the UAV path-line. The operators used on the genotypes are a combination of selection, recombination, and mutation of chromosomes. The chromosomes take the form of B-spline control points, but the cost function is evaluated over the actual B-spline path. After a random initialisation, optionally bounded by limits, initial paths are generated and evaluated. The selection scheme used is an elitist truncation model where a threshold parameter *T* allows some of the ‘children’ showing the best fitness to be chosen as ‘parents’ for the following generation/iteration. Of the top T% solutions, the probability of being chosen as future parents is directly proportional to the solution’s fitness, compared to the average fitness of the selected T%. When T=100%, the selection scheme acts like a roulette scheme, where population members are chosen at random. When T≈10%, it acts like a traditional truncation model whereby the fittest members are selected almost exclusively. Nikolos et al. found 40%≤T≤70% as suitable and sufficient to avoid entrapment in local minima. The results presented are 3D simulations with a cost function that considers proximity to terrain and vehicle capabilities such as minimum path curvature angles and maximum flight height. The computational cost of the process is not discussed; however, the team concludes that the method is feasible under forced constraints and in a limited time period, given the low number of iterations needed to optimise the locations of the B-spline control points.

NSGA-II is a Non-dominated Sorting Genetic Algorithm that was first published by Deb et al. in 2002 as an alternative to traditional multi-objective non-dominated EAs [18]. A solution is said to be non-dominated if no aspects of the cost function can be further improved without worsening other parts of the cost function. Mittal and Deb use the NSGA-II EA and local searches as an offline method of finding paths for a UAV in 3D, considering two main objectives [19]. They define two problem types:

- UAV navigation with no restriction on the path,
- UAV navigation with a requirement for the aircraft to pass through one or more specified points.

Once again, B-splines are used to generate a smooth path and the free-to-move control points are the source of change in the path. Mittal and Deb argue that combining multiple metrics into a single objective optimisation problem holds risks. Firstly, the combination of multiple objectives requires that all objectives must be of the same type (i.e., minimisation or maximisation), and, secondly, that weighting vectors require normalised objectives and mean an implicit user input of comparative importance between objectives. The objective functions considered here are the minimisation of the length of path, and the often contradictory minimisation of risk due to ground proximity. A number of constraints are placed on the problem, including not colliding with the terrain and maintaining smooth changes of direction. Mittal solves the issue of combining objectives by finding *N* non-dominated solutions to each objective and applying a local search procedure to reduce *N* to between 8–10.

Although Mittal and Deb find a way around the implicit user-weighting during the combination of objectives, the process presented serves only to move the user input to the end of the process where the paths must be qualitatively compared. The process is arguably suitable to problems with two objectives, however, any more than that would make the the final user-decision difficult to visualise. The computational time of the presented form was several minutes at the time of publication. Additionally, an automated decision-making process would need to be appended in order to select a single path from the generated candidates.

Wise et al. present a method providing real-time path-planning in tactical scenarios using an evolution-based algorithm [20]. Tested using a high-fidelity UAV simulator, the 2D algorithm finds a quasi-optimal path on demand within a given time window. Two test cases were primarily used; the first with a requirement to orbit a target within a 5% error in radius, and the second to loiter outside of radar detection at a given radius from a known target location. One of the novel aspects of the presented method is the incorporation of a planning horizon. This means that, after an initial offline initialisation, the aircraft has the transit time to the horizon in which to calculate the next segment of the flight path. This approach means that only small sections of the path must be generated at any given stage, making real-time computation possible. The appropriate choice of constraints and cost functions, and some knowledge of the environment, would determine the success of the method in reality. No real-world flights are presented in this work.

More recently, MahmoudZadeh et al. using evolutionary algorithms to solve a rendezvous problem in a cluttered environment for an autonomous underwater vehicle [21]. Xue and Sun also present an evolutionary algorithm in their 2018 paper, using a multi-objective cost function to optimise paths for simulated mobile robots [22]. The use of evolutionary-type methods in these recent publications, despite not relating directly to UAVs, indicate continued relevance of this established optimisation method in the field of robotics and path planning.

### 2.3. Particle Swarm Optimisation

The Particle Swarm Optimisation (PSO) method originates from the social behaviours of animals such as fish and birds [23]. A population of particles is called a swarm, with every particle representing a candidate solution within the search space. The swarm and space exist in *d* dimensions for a problem defined with *d* dimensions. Every particle has a position $x\in R^{d}$ and a velocity $v\in R^{d}$, both randomly initialised within the search space. The particles’ positions are updated based on their velocities with each iteration. The individual velocity of each particle is affected by the previous velocity of the particle, the best ever position held by the particle (‘personal influence’), and the best ever position held by any particle (‘global influence’). The equations of the method tend to follow the form shown in Equations (1) and (2), where *i* is the iteration number, *n* is the particle number, $r_{1}$ and $r_{2}$ are random values between 0 and 1, g is the best ever position of any particle within the swarm, p is the best position occupied by particle *n*, $c_{1}$ and $c_{2}$ are the personal and global influence parameters, respectively, and ω is the ‘inertia’ of the particle:

(1) $$v_{i+1}\left(n\right)=\omega v_{i}\left(n\right)+r_{1}c_{1}\left(p_{i}\left(n\right)-x_{i}\left(n\right)\right)+r_{2}c_{2}\left(g_{i}-x_{i}\left(n\right)\right),$$

(2) $$x_{i+1}\left(n\right)=x_{i}\left(n\right)+v_{i+1}\left(n\right).$$

Salamat and Tonello use a PSO to generate trajectories for quad-rotor UAVs, and compare the performance of the PSO to the standard A* algorithm, Rapidly-exploring Random Tree algorithm, and a GA for the minimum length path in a 2D problem, summarised here [23]. Salamat defines the location of the start and end points, and the number of control points used. The execution time was on the order of 5–7.5 s depending on the specific problem, with swarm sizes of 50–100, 4–5 control points, and between 250–400 iterations. The application of these methods to multi-rotor UAVs is distinctly different to using them for fixed-wing UAVs; however, the comparison of these results is pertinent to the selection of an initial method for trial.

The PSO was first quantitatively compared to a GA by Roberge et al. [16], who conduct a thorough comparison of the methods in a real-time 3D UAV path-planning context. This is one of only a few papers that do not use B-splines for paths; instead, they use line segments, circular arcs, and vertical helices between points. The solutions found were feasible and near-optimal; however, the computation time was proving to be an issue so the team parallelised the algorithms. The result allowed computation times as short as 3.63 s; however, an execution time of 10 s was justified as allowable for online application considering the typical speed of fixed-wing UAVs and the scale of the paths generated. It is important to note here that, although real-time calculation is mentioned, it was only performed in simulation and the computing power required would limit route calculation to being ground-based. With terrain models from GeoBase (Canada), extensive results are presented summarising PSO and GA performance over 60 trajectories in 40 scenarios applied to two fictitious and six real terrain maps.

A statistical *t*-test was carried out on the cost-distributions of the trajectories, and, based on the results, the GA was shown to be preferable. The fixed computation time of 10 s was applied throughout, and both methods had a population size of 256. The difference in result between this paper and Salamat et al. shows how, when applied to different problems, the most preferable algorithm is not easily predictable.

### 2.4. Other Optimisation Methods

Other methods that have been applied to UAVs include the Grasshopper Optimisation Algorithm, which was applied in 3D to solar UAVs by Wu et al. for navigation around congested urban environments while considering the visibility of the sun [24]. Zhang et al. consider the Ant Colony method for UAV path planning, however only in a 2D environment [12].

Alongside SA, the GA method was selected for initial testing due to the promising results presented in the literature. As justified below in Section 3.1, the GA was then chosen for further development and implementation.

### 2.5. Real-Time Navigation

Real-time moth-inspired navigation algorithms have been developed on multi-rotors for finding the source of forest fire plumes by Montes, with further simulations and real-world tests presented by Letheren et al. [2,25]. In some sense, it is similar to the plume sampling discussed in this paper; however, the source of volcanic emissions is known and static, whereas the forest fire source may move rapidly.

In order to better test the performance of the algorithms used, Letheren et al. developed a novel way of modelling the plume for UAV simulations by adding a variable wind-speed to the Holzbecher model [26]. Along with a random plume generation during the execution of the process, a stochastic element was introduced to the modelling of the plume tracker. Purely computational simulations were run in 3D successfully; then, hardware in the loop simulations were used to troubleshoot the real-world system.

Letheren et al. apply the above work to a 3DR IRIS quadcopter UAV, tracking a plume in 2D in real time [2]. The payload of the aircraft is sufficient for the sensor and an additional telemetry unit, with the computation of the algorithm conducted on the ground station. Although this is sufficient for the example presented in this paper, the telemetry links to small UAVs are often not sufficiently reliable when operating at BVLOS range.

## 3. Methodology

### 3.1. Algorithm Selection

A number of algorithms were explored for implementation on-board UAVs as a RTTP, as discussed in Section 2. The GA and SA methods were selected for initial testing, and, while both worked well in 2D, the GA proved superior to SA in initial 3D testing. Considering the work of Nikolos et al. and Roberge et al., the GA was chosen for implementation to the real-world case presented in this paper [16,17]. The purpose of the work presented here is to establish an initial data set for real world on-board RTTP; therefore, the specifics of the optimisation function are secondary to the implementation.

### 3.2. World Space and Trajectory Representation

The representation of the world space includes terrain, start and end locations, and ‘No Go’ zones. NASA’s SRTM terrain data was downloaded at 3-arc second resolution for operations in Guatemala [27]. This means an approximate distance between data points of 90 m at this location on the globe, with each data point representing an average height above mean sea level (AMSL) of the local and neighbouring 1-arc second data. For UAV operation in environments such as this, where proximity to the ground is avoided, an additional safety buffer can be added to the altitude data, making the resolution sufficient for terrain avoidance.

Point locations were described in the following format:

(3) $$Location=\left[\begin{array}{ccc} lat & lon & h \end{array}\right],$$

where *h* represents the altitude of the locations in meters AMSL, referenced to the WGS84 SRTM model. Areas to be avoided were described as ‘No Go’ zones or ‘Obstacles’, limited initially to cylindrical shapes of infinite height. Future work involves combining cylinders to form complex shapes, with the addition of altitude limits. The format of these zones follows:

(4) $$NoGo=\left[\begin{array}{ccc} lat_{1} & lon_{1} & r_{1}\\ lat_{2} & lon_{2} & r_{2}\\ \ldots & \ldots & \ldots \\ lat_{n} & lon_{n} & r_{n} \end{array}\right],$$

where $r_{n}$ represents the radius of zone *n* in meters, centred on $lat_{n}$, $lon_{n}$ (decimal degrees). The trajectory was described as a series of waypoints in the format given in Equation (5), with each waypoint represented by a latitude, longitude, and altitude (metres AMSL):

(5) $$Trajectory=\left[\begin{array}{ccc} lat_{1} & lon_{1} & h_{1}\\ lat_{2} & lon_{2} & h_{2}\\ \ldots & \ldots & \ldots \\ lat_{n} & lon_{n} & h_{n} \end{array}\right],$$

where $h_{n}$ represents the altitude of waypoint *n* in metres AMSL.

### 3.3. Cost Function

The structure of the cost function was partly inspired by Roberge et al., and is made up of *feasibility criteria* and *optimisation criteria* [16]. The former must be satisfied for a route to be possible and the latter define the characteristics of the route. The general equation is given in Equation (6):

(6) $$Cost=\underset{\mathrm{Feasibility}\mathrm{Criteria}}{\underset{︸}{C_{Terrain}+C_{Power}}}\;+\underset{\mathrm{Optimisation}\mathrm{Criteria}}{\underset{︸}{C_{Length}+C_{Altitude}+C_{NoGo}+C_{Climb}}}\;.$$

Each of the terms given in Equation (6) is described below, starting with the term associated with the length of the path:

(7) $$C_{Length}=1-\frac{L_{AB}}{L_{traj}},$$

∴

(8) $$C_{Length}\in \left[0,1\right],$$

where $L_{AB}$ is the straight line distance from A to B, the start and end points of the route, and $L_{traj}$ is the distance of the route in question. The purpose of this criterion is to minimise the length of the trajectory. This criterion tends to zero as the trajectory length decreases.

Keeping the average altitude low helps reduce unnecessary climbing and therefore unnecessary energy expenditure. This was affected by $C_{Altitude}$:

(9) $$C_{Altitude}=\frac{h_{traj}-h_{ABmin}}{T_{max}-h_{ABmin}}$$

set with limits of

(10) $$C_{Altitude}\in \left[0,1\right],$$

where $h_{traj}$ is the average altitude of the waypoints in the trajectory, $T_{max}$ is the maximum altitude of the terrain data for the area, and $h_{ABmin}=min\left[h_{A},h_{B}\right]$, the altitude of the lower of the start and end points. This critereon tends to 1 as average trajectory height tends to $T_{max}$.

While not permitted to enter ‘No Go’ zones, flight through them in this case is physically possible and therefore they are included in the cost function as an optimisation criterion, rather than feasibility criteria:

(11) $$C_{NoGo}=\frac{L_{inNoGo}}{\sum_{i=1}^{n}rad_{i}}$$

set with limits of

(12) $$C_{NoGo}\in \left[0,1\right],$$

where $L_{inNoGo}$ is the length of the route inside ‘No Go’ zones, *n* is the number of obstacles, and $rad_{i}$ is the radius of the ‘No Go’ zone *i*.

In addition to minimising the average altitude of the route, the cost function penalises over-climbing. $C_{Climb}$ aims to minimise the altitude gain of the trajectory, avoiding over climbing:

(13) $$C_{Climb}=1-\frac{h_{B}-h_{A}}{\sum_{i=1}^{n-1}d_{i}},$$

where

(14) $$d_{i}=\left\{\begin{array}{cc} h_{i+1}-h_{i}, & (h_{i+1}-h_{i})>0,\\ 0, & (h_{i+1}-h_{i})<=0 \end{array}\right.$$

and ∴

(15) $$C_{Climb}\in \left[0,1\right],$$

where $h_{A}$ is the altitude of the start location altitude, and $h_{B}$ is the altitude of the end location.

Two feasibility criteria were used; $C_{Terrain}$ penalises trajectories that pass underground, and $C_{Power}$ penalises trajectories with unrealistic climb angles. These components are different to the optimisation components of the cost function by the addition of *P* when the trajectories were not feasible, where *P* is the number of optimisation criteria. In this case, P=4:

(16) $$C_{Terrain}=\left\{\begin{array}{cc} P+\frac{L_{under}}{L_{traj}}, & L_{under}>0,\\ 0, & L_{under}=0, \end{array}\right.$$

so

(17) $$C_{Terrain}\in 0\cup \left[P,P+1\right],$$

where $L_{under}$ is the length of the route underground.

A similar approach was taken to penalising high climb angles, with the length of legs over a given angle summed and if not zero then included:

(18) $$C_{Power}=\left\{\begin{array}{cc} P+\frac{L_{overMax}}{L_{traj}}, & L_{overMax}>0,\\ 0, & L_{overMax}=0, \end{array}\right.$$

so

(19) $$C_{Power}\in 0\cup \left[P,P+1\right],$$

where $L_{overMax}$ is the cumulative length of the legs in the trajectory that exceed a specified climb angle. This value will vary with the aircraft type and atmospheric conditions but was kept constant at 10 degrees for the flights presented in this paper.

### 3.4. Genetic Algorithm

This section describes the implementation of the GA used in this work. The PsuedoCode for the GA is given in Algorithm 1. An initial population is generated with a Gaussian distribution from the midpoint between the start and end locations. A chromosome is formed by arranging the waypoints as shown in Equation (5) and evaluating the cost of that trajectory. Each chromosome within the generation is manipulated with crossover or mutation operators, and in this version only operations that have a positive effect on the fitness of the chromosome are accepted as children. Once the entire generation has been operated on, it is sorted according to the fitness of the children solutions and the lowest quarter is replaced with new routes. The new genomes are the result of a crossover between one of the top quarter solutions and a random new solution, and are made chromosomes by evaluating the cost function. This was found to balance exploration and exploitation of the design space through the optimisation process. 

**Algorithm 1:** Genetic Algorithm PseudoCode

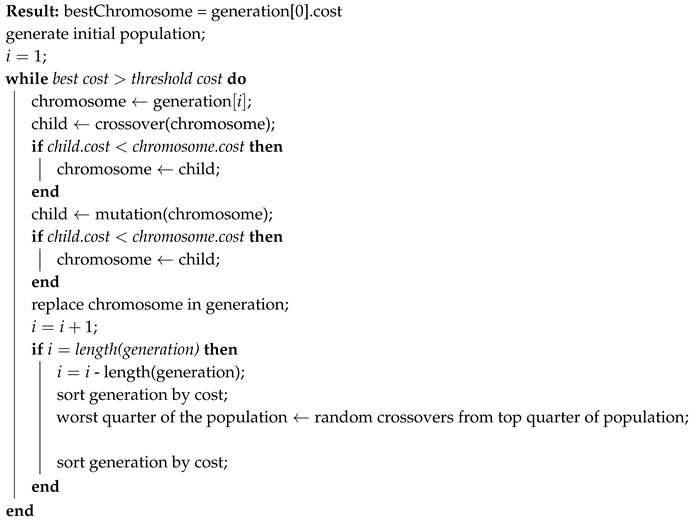



The purpose of this code is to run in real time, therefore the threshold cost was tuned for the real world space in which the aircraft would fly. Software-in-the-loop simulations meant tests could be conducted in a safe and repeatable manner, and a threshold cost of 1.5 was found to be suitable for initial flight testing.

#### 3.4.1. Chromosome Generation

New trajectories are generated by creating between 2 and 8 waypoints joining the given start and end locations together. For a given trajectory, an iterative waypoint generation process is conducted where the waypoints have a Gaussian distribution centred on the halfway point between the start and end locations, with a standard deviation of $\frac{L_{AB}}{2}$. The altitude is assigned by adding or subtracting an appropriate amount from the preceding waypoint altitude, using a flight path angle that is (uniformly) randomly selected from a prescribed range, according to the aircraft’s limits.

A population size of 10 was used for the results shown in this paper. A larger population size weights exploration over exploitation, generating too many new chromosomes and not operating on them enough. Exploitation was weighted over exploration with population sizes <10, leading to local minima and sub-optimal results.

#### 3.4.2. Crossover Operation

**Figure 2:**
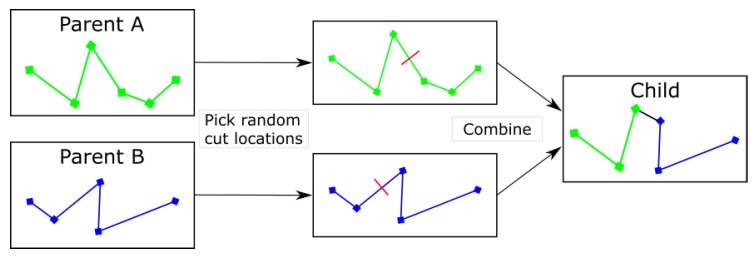
The function of a Genetic Algorithm crossover operator in a 2D path optimisation example.

The crossover operation used in this version of the GA required two parent routes. A cut point for each parent was randomly chosen and the routes added together. The altitude of the waypoints remained consistent throughout, making the operation similar to a two-dimensional trajectory or path crossover function (Figure 2).

#### 3.4.3. Mutation Operation

**Figure 3:**
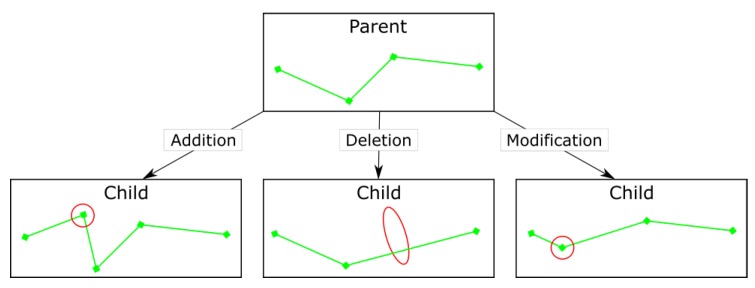
Possible functions of a Genetic Algorithm mutation operator in a 2D path optimisation example.

One of three types of mutation was randomly chosen to be attempted: waypoint (WP) addition, deletion, and modification (Figure 3). The first two involve the addition or deletion of a WP at a random point between the start and end locations. The modification operator involves effectively combining the two, first selecting a random WP then assigning it a new set of coordinates and altitude using the Gaussian waypoint generator mentioned above.

## 4. Implementation

The real world implementation of the RTTP can be split into three main sections: hardware, mission control, and communications; each of which is discussed in this section.

### 4.1. Hardware

The flights presented here were conducted with a Skywalker X8 airframe, manufactured by Skywalker (China). The airframe was assembled at the University of Bristol and fitted with long-range equipment for operation over Fuego. A summary of the aircraft’s specifications is given in Table 1, and further information can be found in [28].

The RTTP script was run on a Raspberry Pi 3 B+ (Raspberry Pi, Pencoed, UK), mounted in the payload bay of the aircraft and powered by the aircraft flight batteries. The Raspberry Pi had a serial connection to the Pixhawk autopilot, which was used for receiving and sending MAVLink messages.

### 4.2. Mission Control

The mission control program is responsible for running the main loop, as described in Algorithm 2. Once power is turned on and all systems are initiated, the algorithm waits for the aircraft mode to be turned to ‘Guided’ mode. This is a positive action from the pilot and control can always be regained by the pilot by switching out of ‘Guided’ mode. Conventional operations are flown in ‘Auto’ mode, following a pre-set mission plan. ‘FBWA’ mode has also been used for pilot-guided first person view (FPV) flight for accurate plume interception. 

**Algorithm 2:** RTTP Mission Control Algorithm

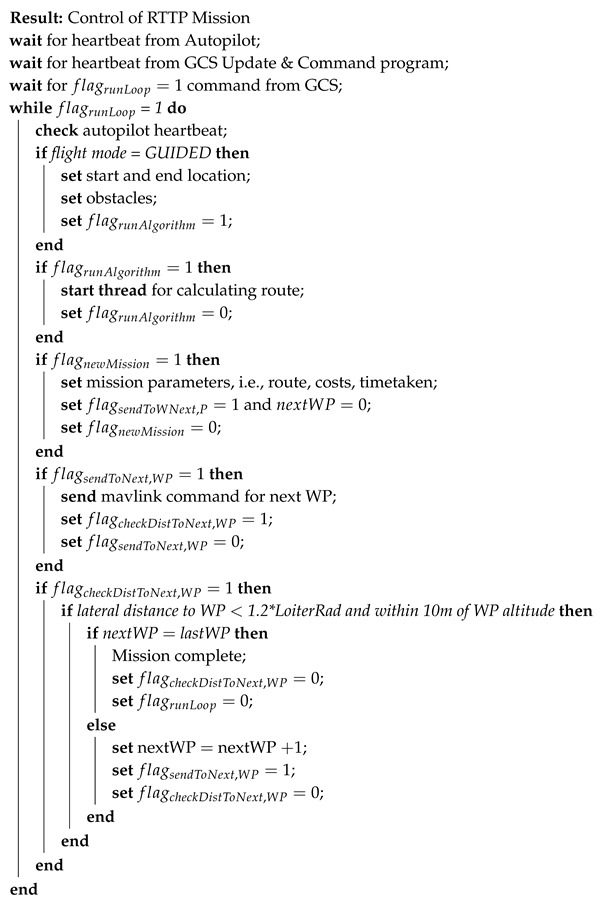



In addition to the main loop, the mission control script runs two threads. The first is responsible for handling MAVLink messages, a standard message protocol used by the ArduPilot software, and the second is responsible for running the optimisation algorithm when required. The version of the RTTP that is presented here generated just one route per flight, when the mission control script demanded a new route. Future implementation of this method would involve modified criteria for a new mission to be calculated, reassessing the trajectory either on a regular basis or when new information is obtained.

### 4.3. Ground Station Update and Command

It is vital to have a line of communication between the GCS, where the operators make key decisions about the aircraft and mission progress, and the RTTP algorithm. There are two key components to this: updates regarding the progress of the RTTP, and sending commands to the RTTP. The progress of the aircraft was monitored from this program, with the pilot taking control when appropriate depending on the mission progress and payload requirements.

## 5. Results

This section presents results from four RTTP flights and a number of conventional flights. A comparison is made, and the cost function applied to the routes of the conventional missions. The purpose of these results is to demonstrate that it is possible to fly a fixed wing UAV mission using an on-board RTTP, and to compare these initial flight tests to conventionally navigated flights in the same area with similar parameters.

Plots in this section show just the climbing phase flight path as this is when the RTTP was used. Time spent monitoring the volcano, and the descent phase of the flight, have been removed for clarity. The full flight logs are provided as supplementary information accompanying this paper.

### 5.1. RTTP Flights

The four RTTP flights presented here took place in March 2019. Every flight involved calculation of a new and valid route; however, some were not flown to completion. One successful and one failed flight is presented for a case with no obstacles, and one successful and one failed flight is presented for a case with two obstacles.

In some cases, the autopilot has been known to climb at the maximum possible climb angle to reach the altitude required, rather than follow the flight path linking consecutive waypoints. Manually generated mission plans can take this into account; however, the behaviour demonstrated by the UAV during the RTTP flights showed this same approach to climbing. This naturally makes the flight less efficient, and in some previous cases has been known to cause extremely high-drag flight configurations.

Initially, the start location, end location, and any obstacles were set via the GCS Update & Command program. The aircraft was then switched to ‘Guided’ mode and would circle at that location until a solution was found. Upon receiving a new mission, the UAV would fly to the start location before following the calculated route. RTTP parameters such as threshold solution cost and population size were tuned using Hardware In The Loop simulations, and kept constant across all four flights in order to maintain comparability.

#### 5.1.1. Flight A: 31st March 2019

This was the first flight to use the RTTP in Guatemala. A solution was found rapidly and the aircraft was sent to the start location. In order to progress to the second waypoint, the aircraft had to be within a threshold radius of the first waypoint. Parameters on-board the aircraft meant the UAV circled the waypoint with a radius larger than this threshold, effectively halting progress along Algorithm 2. This was overridden via the GCS Update & Command Program, and the UAV was sent to the next RTTP waypoint; however, during the first leg, the operational decision was made to switch the aircraft to ‘Auto’ mode and continue the flight in a conventional manner.

In order to combat the issue of not reaching the threshold distance from the waypoint, the Mission Control code was modified to increase the threshold distance. Flight A is included in the results in part because it represents the first use of an on-board RTTP generating and following a BVLOS flight plan, despite it not reaching the end location.

**Figure 4:**
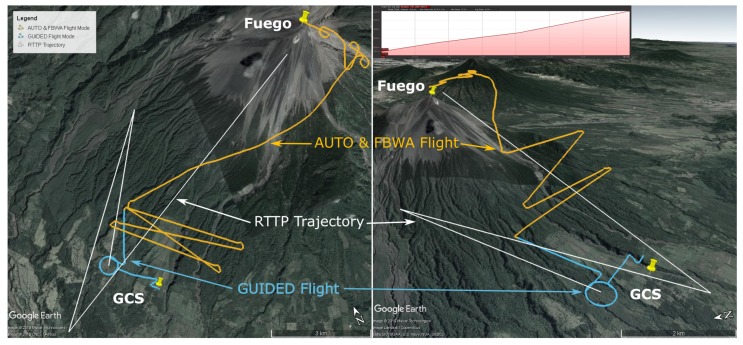
Flight A data: top down (left) and 3D (right) maps showing the Real Time Trajectory Planner solution (white) and the actual flight path (Guided: Blue, Auto/FBWA: Orange) in Google Earth [29]. The elevation profile of the RTTP Trajectory is inset (top, right).

The parameters of the RTTP solution are given in Table 2 and a plot of the trajectory is shown in Figure 4.

Note the inset gradient of the RTTP trajectory in Figure 4, with an average gradient of 7 degrees. The profile is relatively consistent throughout the trajectory with a maximum gradient of 9.7 degrees and no excess climbing.

#### 5.1.2. Flight B: 31st March 2019

**Figure 5:**
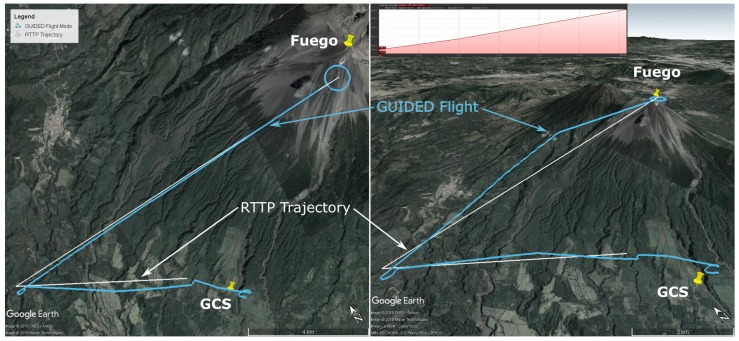
Flight B data: top down (left) and 3D (right) maps showing the Real Time Trajectory Planner solution (white) and the actual flight path (Guided: Blue) in Google Earth [29]. The elevation profile of the RTTP Trajectory is inset (top, right). For scale, the length of the longest leg here is 11.3 km.

This flight was the first successful implementation of Algorithm 2. The aircraft reached the target location in 21 min 13 s with 51.1% battery capacity remaining. A proper comparison of these figures will follow in Section 5.2. Flight parameters are presented in Table 3 and the flight path and RTTP trajectory are shown in Figure 5. For scale, the length of the longest leg in Figure 5 is 11.3 km.

Note the inset gradient of the RTTP trajectory in Figure 5, with an average gradient of 8.1 degrees. The profile is relatively consistent throughout the trajectory with a maximum gradient of 10.2 degrees and no excess climbing.

#### 5.1.3. Flight C: 1st April 2019

**Figure 6:**
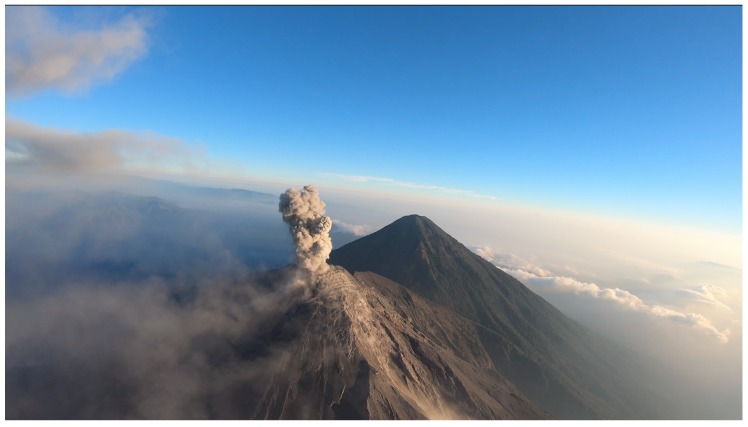
A typical eruption from Fuego (approx 3800 m AMSL) as seen from the on-board camera during an ash-sampling mission. The Ground COntrol Station is at an altitude of 1137 m AMSL.

**Figure 7:**
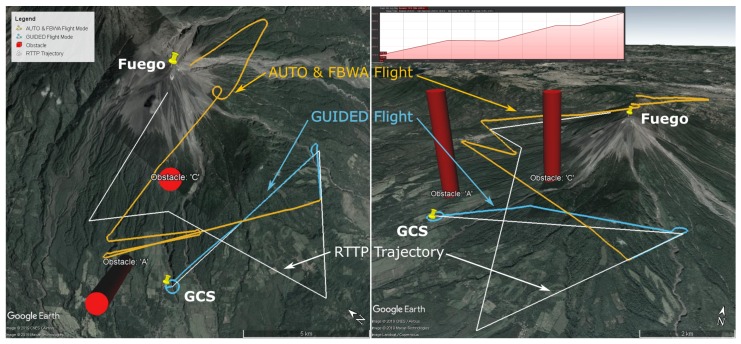
Flight C data: top down (left) and 3D (right) maps showing the Real Time Trajectory Planner solution (white) and the actual flight path (Guided: Blue, Auto/FBWA: Orange) in Google Earth [29]. Obstacles are shown by red cylinders, and the elevation profile of the RTTP Trajectory is inset (top, right).

This flight was the first to use the RTTP with obstacles over Fuego in Guatemala. A valid route was generated; however, it was only followed in part before switching to the conventional flight path. A desirable eruption occurred which led to the operational decision to end the RTTP phase of the flight in order to sample the plume as immediately as possible. An example of such an eruption is pictured in Figure 6. The route parameters are given in Table 4 and the flight path and route are shown in Figure 7.

Note the inset gradient of the RTTP trajectory in Figure 7, with an average gradient of 7.9 degrees. The profile has a maximum gradient of 9.3 degrees with 38 m of excess climbing.

#### 5.1.4. Flight D: 2nd April 2019

**Figure 8:**
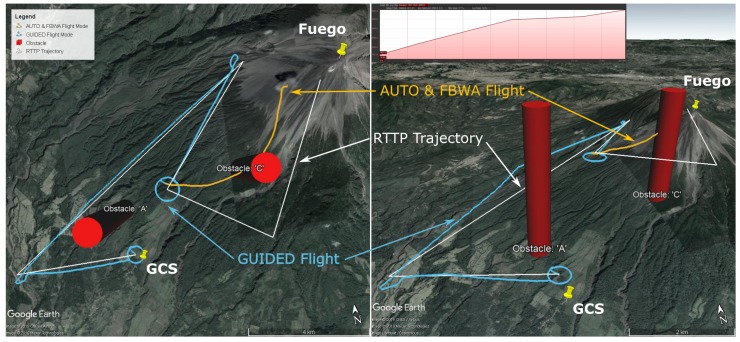
Flight D data: top down (left) and 3D (right) maps showing the Real Time Trajectory Planner solution (white) and the actual flight path (Guided: Blue, Auto/FBWA: Orange) in Google Earth [29]. The elevation profile of the RTTP Trajectory is inset (top, right).

This flight was the first successful implementation of Algorithm 2 with two ‘No Go’ zones in place. The aircraft reached the penultimate waypoint, at approximately summit altitude, in 25 min 41 s, with 41.8% battery capacity remaining. The UAV was not allowed to reach the final RTTP waypoint because there was a plume a short distance west of the path between the final two waypoints, and control was re-assumed in order to intercept the plume for sampling purposes. Flight parameters are presented in Table 5 and the flight path and RTTP trajectory are shown in Figure 8.

Note the inset gradient of the RTTP trajectory in Figure 8, with an average gradient of 6.2 degrees. The profile starts and ends with a steeper section, with a maximum gradient of 10 degrees and no excess climbing.

### 5.2. Conventional Flights

Two mission plan types have previously been used, one of which will be compared to the RTTP results presented here. The first of these involved a spiral climb, and the second, which was moved to because it is significantly more efficient, involved straight-leg climbs. For the sake of comparison, the end location may not be the same exact location as in the RTTP routes shown above; however, it is in the immediate vicinity of Fuego’s summit so the values are comparable.

For the sake of valid comparison, only flights matching certain criteria are presented in this section; the aircraft flown for both RTTP and ‘normal’ flights is the same, with consistent payload and equipment configurations. The weather will naturally have varied between flights, and the mass of the aircraft was increased for the RTTP missions by the addition of the Raspberry Pi and associated cables (approx. 50 g).

**Figure 9:**
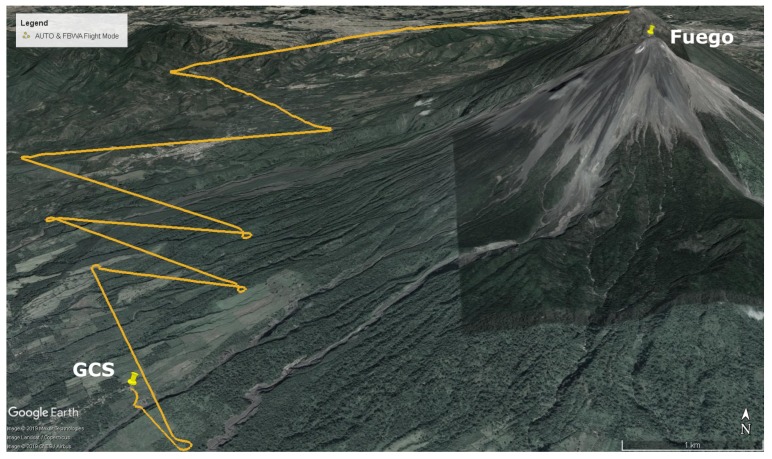
Example of a conventional leg climb route, developed over a series of trips and flights.

A conventional straight leg climb method was developed through trial and error, significantly reducing the energy required to reach both summit altitude and the summit area compared to initial attempts. An example of this type of climb is shown in Figure 9. Key parameters of these ‘standard’ mission types are presented in Table 6, alongside data from each of the RTTP flights.

Airspeed data (Table 6) show that conventional mission plan flights had a higher average airspeed but also a higher standard deviation when compared to RTTP flights. The lower airspeeds of the RTTP flights can be explained by the steep, inefficient climb angle used by the autopilot on these flights. Comparing airspeed and calculated ground speed (grounddistance/flighttime), the effect of the wind around Fuego is clear; for example, the average ground-speed for conventional missions was 17.4 m/s compared to 24.5 m/s airspeed.

## 6. Discussion

As mentioned in Section 5.1, there is a behaviour shown by the autopilot in some instances which causes the UAV to climb at its maximum climb rate until the target altitude has been reached. The RTTP results presented show that this behaviour was present while following the RTTP trajectory in Guided mode. The effect of this on the flight path is particularly visible in Figure 5 by comparing the top–down view and the three-dimensional view. Climbing in this manner induces excessive drag and slows the aircraft down, compared to a gradual climb along the flight path which arrives at the target altitude upon reaching the waypoint. An example of a conventionally generated trajectory that takes this behaviour into account can be seen by the Orange AUTO/FBWA flight track in Figure 4. The real world efficiency of the RTTP flights presented here is therefore worse than can be expected. Should a change be made to the behaviour of the autopilot in cases such as these, the effect on RTTP flights would be to increase their efficiency, both in terms of raw data and comparison to conventionally generated mission trajectories.

Flight A and Flight B represent RTTP paths that do not take into account any obstacles, and are compared to data from conventional missions in Table 6. While conventional missions have been carefully designed to climb near the GCS and spend much of the approach to Fuego at summit and plume altitude, the RTTP trajectories are not restricted to these constraints. RTTP routes A and B average a trajectory length 34% shorter than conventionally planned missions, with cost function values of approximately half and a significantly quicker transit time. The only RTTP route flown to completion here is Flight B and the battery capacity remaining at the end location, despite the shortcomings of the autopilot’s behaviour in Guided mode, represents a 2% saving. With a smoother implementation of the climb by the autopilot, this small saving is likely to increase significantly.

While the obstacles used for Flight C and Flight D (Figure 7 and Figure 8 respectively) do not represent anything tangible in reality, the RTTP was tested with them for completeness. The conventional mission plan (Figure 9) was not designed with these hypothetical obstacles in mind, hence Flights C and D are not truly comparable to conventional mission data. Table 6 shows a an average RTTP trajectory length that is slightly shorter (7%) than planned missions, but a slightly longer transit time and and 7% more battery consumption. The shorter missions with longer transit time indicates environmental factors such as wind affecting the true ground speed of the aircraft. Future versions of the RTTP should incorporate wind speed and direction, using environmental factors to further increase overall flight efficiency. The cost function for the RTTP routes is 40% less than the conventional routes; however, the function was tuned for the RTTP so it cannot be considered a pure form of comparison. The autopilot’s climbing behaviour partly explains the comparative inefficiency of Flight D and, as is true for all the RTTP data presented here, further development and testing is required before significant efficiency savings can be claimed.

An RTTP removes the need to manually develop a conventional flight plan. Tuned correctly, and with dynamic assessment of conditions and re-routing, this would avoid the typical trial-and-error approach to flying in new locations. The conventional flight plan that is shown in Figure 9 took several trips and over 20 flights to develop, and the RTTP trajectories shown here are comparable according to many of the parameters in Table 6. Not only might an RTTP offer energy savings in-flight, but also increase operational efficiency in the more general case of operating at new locations.

## 7. Conclusions

According to the data presented in Table 6, some efficiency gains can be expected by using the RTTP; however, the system as presented does not offer significant gains. The results presented in this paper demonstrate that it is not only possible to fly a RTTP on-board a UAV, but that, in some cases, it can increase the efficiency of the platform. Further work in this area would involve refinement of the optimisation algorithm, bench-marking optimisation performance, use of complex dynamic obstacles, and comparison to non-GA methods such as PSO or other, newer, methods. The implementation algorithm could be further streamlined and the behaviour of the autopilot when carrying out the RTTP mission must be amended so that unnecessary drag in the climb is minimised. Inclusion of the wind speed and direction in the RTTP should lead to more efficient climb paths, increasing the average RTTP airspeed while reducing the battery capacity used. Combining these areas of future work can only improve the efficiency of the aircraft in environments such as that presented in this paper. As defined in Section 1, the level of UAV automation is increased by allowing the systems on-board the aircraft the authority to produce and follow a trajectory, with human input required as a fail-safe rather than a positive action for the RTTP route to be followed.

The results presented here prove it is possible to use an RTTP for conducting UAV missions. The circumstances surrounding the flights presented, such as the autopilot’s approach to following RTTP missions in ‘Guided’ mode, suggest that, with further development, significant energy savings would be made using an on-board RTTP. Additionally, these early tests generated routes comparable to conventional flight plans, which took significant time to refine. Further development of the RTTP is therefore also likely to result in a significant reduction of the time taken to fly successful and efficient missions after arriving at new sites.

## Figures and Tables

**Table 1:** Skywalker X8 Specifications.

Parameter	Value
Flight Time	45 min
Take-off mass	4.2 kg
Wingspan	2.1 m
Battery	2 × 8000 mAh, 14.8 V LiPo
Motor	AXi 4120/14,660 kV
Speed Controller	Jeti Adv 77 Pro Opto
AutoPilot	Unmanned Tech PixHawk, ArduPlane 3.7.1
Pilot Link	DragonLine V3 Adv (433 MHz)
Telemetry Link	RFDesign 868+ (868 MHz)
Video Link	ImmersionRC 700 mW (2.4 GHz)

**Table 2:** Flight A Real Time Trajectory Planner parameters.

Parameter	Value
Obstacles	None
Number of Iterations	2
Time Taken	5.15 s
Worst Cost	74.67
Best Cost	1.196

**Table 3:** Flight B Real Time Trajectory Planner Parameters.

Parameter	Value
Obstacles	None
Number of Iterations	14
Time Taken	28.09 s
Worst Cost	65.70
Best Cost	1.126

**Table 4:** Flight C Real Time Trajectory Planner Parameters.

Parameter	Value
Obstacles	Two
Number of Iterations	39
Time Taken	167.61 s
Worst Cost	53.98
Best Cost	1.343

**Table 5:** Flight D Real Time Trajectory Planner Parameters.

Parameter	Value
Obstacles	Two
Number of Iterations	73
Time Taken	162.60 s
Worst Cost	58.99
Best Cost	1.345

**Table 6:** Comparison of flights, with Conventional Mission data presented as Mean/StdDev, n=10.

Parameter	Conventional Mission	RTTP A	RTTP B	RTTP C	RTTB D
Distance (meters)	25,392/2603	18,319	15,301	25,421	21,925
Time (mins:secs)	24:19/03:40	n/a	21:13	n/a	25:41
Capacity Used (mAh)	8101/709	n/a	7828	n/a	9320
Route Cost (no Obstacles)	2.253/0.056	1.196	1.126	n/a	n/a
Route Cost (Obstacles)	2.649/0.448	n/a	n/a	1.343	1.390
Airspeed: Mean (m/s)	24.5/0.6	21.8	21.2	21.7	22.6
Airspeed: Std Dev (m/s)	4.6/0.4	3.8	4.0	3.4	3.9

## Supplementary Materials

The following are available online at https://www.mdpi.com/1424-8220/19/19/4085/s1: Flight A Autopilot Logs, Flight B Autopilot Logs, Flight C Autopilot Logs, Flight D Autopilot Logs.

## Abbreviations

The following abbreviations are used in this manuscript:

UAV	Unmanned Aerival Vehicle
RTTP	Real Time Trajectory Planner
GCS	Ground Control Station
BVLOS	Beyond Visual Line of Sight
VLOS	Visual Line of Sight
CDR	Conflict Detection and Resolution
SA	Simulated Annealing
GA	Genetic Algorithm
EA	Evolutionary Algorithm
PSO	Particle Swarm Optimisation
WP	Waypoint
AMSL	Above Mean Sea Level
FPV	First Person View
